# Estimated Changes in Insulin Prices and Discounts After Entry of New Insulin Products, 2012-2019

**DOI:** 10.1001/jamahealthforum.2023.1430

**Published:** 2023-06-16

**Authors:** Sean Dickson, Nico Gabriel, Walid F. Gellad, Inmaculada Hernandez

**Affiliations:** 1 West Health Policy Center, Washington, DC; 2 Division of Clinical Pharmacy, University of California, San Diego, Skaggs School of Pharmacy and Pharmaceutical Sciences, La Jolla; 3 Division of General Internal Medicine, University of Pittsburgh School of Medicine, Pittsburgh, Pennsylvania; 4 VA Pittsburgh Healthcare System, Pittsburgh, Pennsylvania

## Abstract

**Importance:**

Despite the political salience of insulin prices, no study to date has quantified trends in insulin prices that account for manufacturer discounts (net prices).

**Objective:**

To describe trends in insulin list prices and net prices faced by payers from 2012 to 2019 and estimate changes in net prices after the 2015 to 2017 entry of new insulin products.

**Design, Setting, and Participants:**

This longitudinal study included an analysis of Medicare, Medicaid, and SSR Health drug pricing data from January 1, 2012, to December 31, 2019. Data analyses were performed from June 1, 2022, to October 31, 2022.

**Exposures:**

US sales of insulin products.

**Main Outcomes and Measures:**

Net prices faced by payers were estimated for insulin products as list prices minus manufacturer discounts negotiated in commercial and Medicare Part D markets (ie, commercial discounts). Trends in net prices were evaluated before and after the entry of new insulin products.

**Results:**

Net prices of long-acting insulin products increased at an annual rate of 23.6% from 2012 to 2014 but decreased at an annual rate of 8.3% after the introduction of insulin glargine (Toujeo and Basaglar) and degludec (Tresiba) in 2015. Net prices of short-acting insulin increased at an annual rate of 5.6% from 2012 to 2017 but then decreased from 2018 to 2019 after the introduction of insulin aspart (Fiasp) and lispro (Admelog). For human insulin products, which did not experience entry of new products, net prices increased at an annual rate of 9.2% from 2012 to 2019. From 2012 to 2019, commercial discounts increased from 22.7% to 64.8% for long-acting insulin products, from 37.9% to 66.1% for short-acting insulin products, and from 54.9% to 63.1% for human insulin products.

**Conclusions and Relevance:**

In this longitudinal study of US insulin products, results suggest that insulin prices substantially increased from 2012 to 2015, even after accounting for discounts. The introduction of new insulin products was followed by substantial discounting practices that lowered net prices faced by payers.

## Key Points

**Questions:**

What are the trends in net prices for insulin products, and did those trends change after the introduction of new insulin products, generating savings?

**Findings:**

In this longitudinal study of 2012 to 2019 drug pricing data, after accounting for discounts, net prices of long-acting insulin products faced by payers increased by a mean of 23.6% per year from 2012 to 2014 but decreased by 8.3% from 2015 to 2019, coinciding with the introduction of 3 new long-acting insulin products.

**Meaning:**

The entry of new insulin products was followed by decreases in net prices, which lowered spending on long-acting insulins after 2015.

## Teaser

This longitudinal cross-sectional study investigates changes in insulin prices and discounts after entry of new insulin products from 2012 to 2019.

## Introduction

Although insulin was first commercially manufactured in 1923,^1^ affordability has remained a significant challenge for some patients, with 25% of patients using insulin reporting cost as a barrier to access.^1,2,3,4^ Mean list prices of common long-acting insulin products increased by 42.5% between 2014 and 2019,^5^ helping foment insulin prices as a political issue. A series of congressional investigations targeted insulin prices while manufacturers continued to argue that insulin prices are actually decreasing due to confidential discounts.^6^ These discounts, which we refer to as “commercial discounts,” are negotiated between pharmaceutical manufacturers and pharmacy benefit managers in the form of rebates for Medicare Part D and private insurance plans. Commercial discounts differ from mandatory discounts required by statute under the Medicaid and 340B programs; commercial discounts do not directly reduce patient cost sharing, nor do they reduce the prices faced by uninsured individuals.^7^

Increasing commercial discounts likely result from competition among the legacy insulin brands as well as the introduction of new products. From 2015 to 2017, 5 new insulin products were introduced. In 2015, the long-acting insulin market saw the approval of a high-strength branded insulin glargine (Toujeo)^8^; insulin degludec (Tresiba), an ultralong-acting basal insulin^9^; and biosimilar insulin glargine (Basaglar).^10^ In the fast-acting market, Novo Nordisk introduced a new branded formulation of insulin aspart (Fiasp) in 2017.^11^ The first fast-acting biosimilar insulin, insulin lispro (Admelog), entered the same year.^12^ Basaglar and Admelog are not true biosimilars because they were approved when insulin was still considered a drug and not a biologic, but we follow convention and call them biosimilars to recognize their overall relationship with branded products; neither is interchangeable with its branded counterpart.^13^ As a result, they were treated as branded products by insurers when managing formularies and negotiating discounts (unlike generics or interchangeable biosimilars, which negotiate discounts with pharmacies because they can be substituted for the branded product). In 2020, the US Food and Drug Administration redesignated insulin from a drug to a biologic, allowing for the introduction of interchangeable biosimilar products.^14^ As of August 2022, only 1 interchangeable biosimilar insulin has been approved (insulin glargine-yfgn [Semglee]).^15^

Although previous work has examined changes in list prices of branded drugs after generic drug introduction, there is little characterization of changes in net prices of brand-name drugs in response to new competition.^16,17^ Research has been hampered by difficulty in calculating drug prices after accounting for commercial discounts negotiated between manufacturers and payers. Although a previous investigation examined trends in manufacturer net revenue for insulin products from 2014 to 2018, estimates were reported as mean values across 32 insulin products regardless of insulin type and did not account for mandatory government discounts under the 340B Drug Discount Program or the Medicare Part D coverage gap.^18^

To overcome the limitations of previous estimates of net prices, we applied a recently developed method for estimating commercial discounts.^19^ We estimated changes in insulin net prices faced by payers from January 1, 2012, to December 31, 2019, noting the introduction of new insulin products from 2015 to 2017.

## Methods

### Study Sample

In this longitudinal study, we categorized insulin products available from 2012 to 2019 into 3 groups: (1) long-acting insulin analogues, including insulin glargine (Lantus, Toujeo, and Basaglar), insulin detemir (Levemir), and insulin degludec (Tresiba); (2) short-acting insulin analogues, including insulin lispro (Humalog and Admelog) and insulin aspart (Novolog and Fiasp); and (3) human insulin products, including Novolin and Humulin. Insulin products were categorized at the product level instead of the national drug code level because net sales data are reported only at the brand-name level. Therefore, we did not separately categorize formulations of a product (eg, Humulin N vs Humulin Mix).

### Data Sources

We used 5 data sources: (1) net sales and total units from SSR Health; (2) Medicare Part B and Part D 5% claims; (3) Medicare Part D prescriber use files; (4) Medicare and Medicaid spending dashboards; and (5) the 340B covered entity database. The institutional review board at the University of California, San Diego deemed this study exempt from the need for informed consent because only deidentified data were used in analyses. Data analyses were performed from June 1, 2022, to October 31, 2022. This study followed the Strengthening the Reporting of Observational Studies in Epidemiology (STROBE) reporting guideline.

### Estimates of Prices, Discounts, and Market Share

List price was estimated as mean reimbursement per 100 insulin units in Medicare Part D and was calculated using all national drug codes within a product.^19^ We used the mean Part D reimbursement to account for changes in list price throughout the year as annual mean Part D price volume-weights sales at the various list prices. Commercial discounts were calculated in accordance with a published method that isolates statutory Medicaid and 340B discounts (provided to certain federally designated hospitals and clinics) and Part D coverage gap discounts from confidentially negotiated commercial discounts, as described in the eAppendix in Supplement 1.^19^ Our method also accounts for the cap on Medicaid and 340B statutory discounts. In brief, we estimated total discounts for a product as the difference between gross and net sales. Then total discounts were allocated into statutory discounts to the Medicaid and 340B programs, calculated with statutory formulas and accounting for the “best price” provision; Part D coverage gap discounts, calculated with claims data; and commercial discounts negotiated between manufacturers and payers.^19^ Net price was estimated as list price minus commercial discount and represents the price faced by Part D and private insurance plans after rebates. Market share was calculated as the proportion of insulin units sold in the group health insurance and Medicare Part D markets that were accounted for by each product. These units were estimated by subtracting Part B, Medicaid, and 340B units from total units. List prices, net prices, and commercial discounts were all reported per 100 insulin units to account for variation in product strength and are expressed in nominal dollars. To mitigate how inventory variation may have affected the data, prices and discounts of new products were not reported for the first year after market entry (drugs approved in the first half of a calendar year) or 2 years after market entry (drugs approved in the second half of a calendar year).

The 2019 reports filed by Eli Lilly bundled sales for branded Humalog and the authorized generic drug launched in March 2019. To avoid an overestimation of commercial discounts in 2019 for Humalog, we subtracted units and sales for the authorized generic drug from those of Humalog, as explained in the eAppendix in Supplement 1.

## Results

### Long-Acting Insulin Analogues

#### Class Trends

List prices of long-acting insulin products increased from a mean of $13.45 per 100 insulin units in 2012 to $29.15 in 2019 at an annual rate of 12.3% (Table). The mean net price of long-acting insulin products faced by payers increased from $10.40 in 2012 to $15.88 in 2014 at an annual rate of 23.6% and then decreased to $10.27 by 2019 at an annual rate of −8.3%, resulting in a growth rate of 0.9% across the study period. The decrease observed after 2015 was explained by an almost 3-fold increase in commercial discounts. Across the study period, commercial discounts increased from 22.7% to 64.8%.

**Table.** Mean Annual Change in List and Net Prices by Insulin Class^a^

Price type	Long-acting insulin analogues	Short-acting insulin analogues	Human insulin products
List, mean (SD), $			
2012	13.45	14.70	5.85
2019	29.15	33.34	12.93
Annual change, %	12.3	12.7	12.3
Net, mean (SD), $			
2012	10.40	9.13	2.64
2019	10.27	11.31	4.76
Annual change, %	0.9	3.4	9.2
Discount, mean (SD), $			
2012	3.04	5.57	3.21
2019	18.85	22.03	8.16
Annual change, %	34.4	22.6	14.6
2012 Discount as % list price, %	22.7	37.9	54.9
2019 Discount as % list price, %	64.8	66.1	63.1

^a^ Outcomes are expressed per 100 insulin units. Mean list price, net price, and discount were estimated as a weighted mean of the list price, net price, or discount of each product using the relative market share of each product as a weight. Net prices represent the price faced by Part D and private insurance plans after rebates.

#### Product-Specific Trends

**Figure 1.**
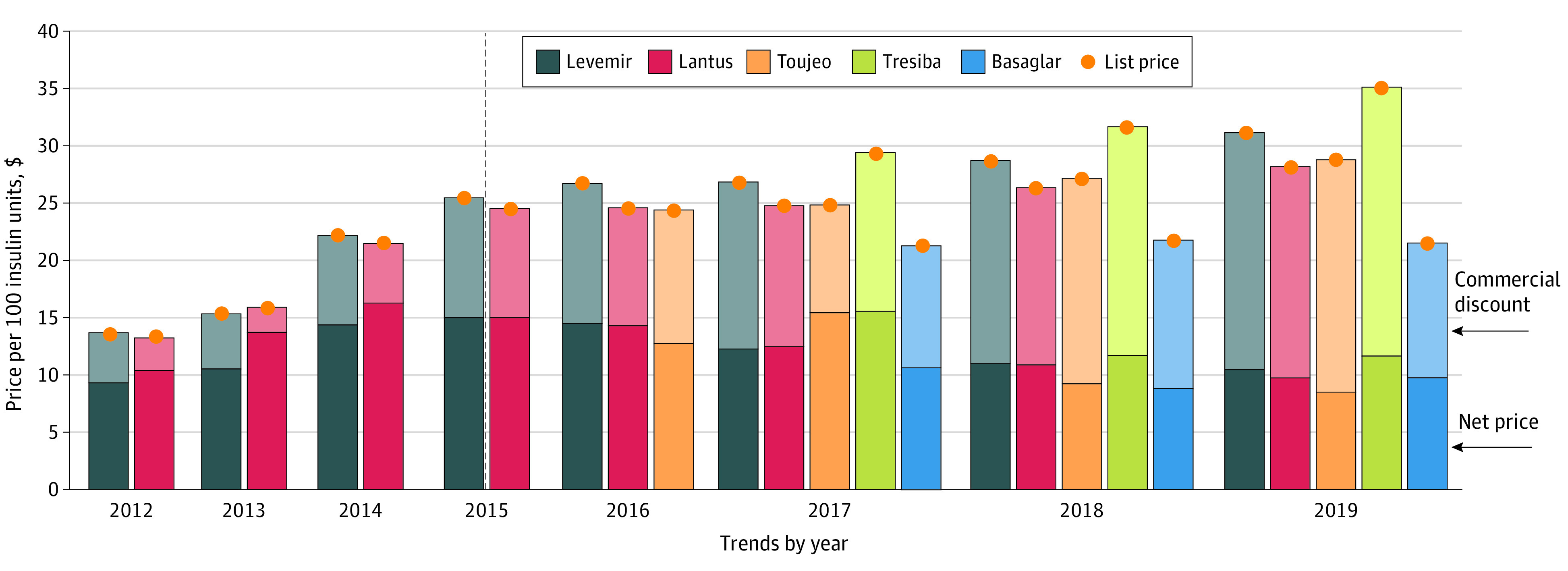
Trends in List Prices, Net Prices, and Discounts for Long-Acting Insulin Analogues List prices were estimated as mean reimbursement rates in Medicare Part D per 100 insulin units. Net prices were estimated as the difference between list prices and commercial discounts and represent the prices faced by Part D and private plans after rebates. The insulin glargine Toujeo was approved in February 2015, insulin degludec (Tresiba) in September 2015, and the insulin glargine Basaglar in December 2015. As explained in the Methods, prices and discounts of new products are not reported for the first year after market entry (drugs approved in the first half of a calendar year) or 2 years after market entry (drugs approved in the second half of a calendar year) to mitigate how inventory variation may have affected the data, which is why data for Toujeo are first reported in 2016 and data for insulin degludec and Basaglar are first reported in 2017. Outcomes are expressed per 100 insulin units. The dashed line indicates approval for Toujeo, insulin degludec, and Basaglar.

The list price ($24.65) of the new product Toujeo, introduced in 2015, closely matched the 2016 list price ($24.71) of the originator Lantus; however, its net price was slightly lower ($12.92 for Toujeo vs $14.56 for Lantus) (Figure 1). The list and net prices of Tresiba were higher than the list and net prices of Lantus and insulin detemir throughout the study period. Basaglar, the product that is bioequivalent to Lantus, had a lower list price than Lantus ($21.50 vs $24.87) in 2017. The net price of Basaglar ($10.92) was slightly lower than the net price of Lantus ($12.79) in 2017, but the net price of Lantus was reduced to match that of Basaglar by 2019 ($9.95 vs $9.99, respectively).

### Short-Acting Insulin Analogues

#### Class Trends

The mean list price of short-acting insulin products increased at an annual rate of 12.7% (Table). The mean net price of short-acting insulin products faced by payers increased at an annual rate of 5.6% from 2012 to 2017 but then decreased from 2018 to 2019, resulting in an annual net growth of 3.4% across the study period. Mean commercial discounts increased from 37.9% of list price in 2012 to 66.1% in 2019.

#### Product-Specific Trends

**Figure 2.**
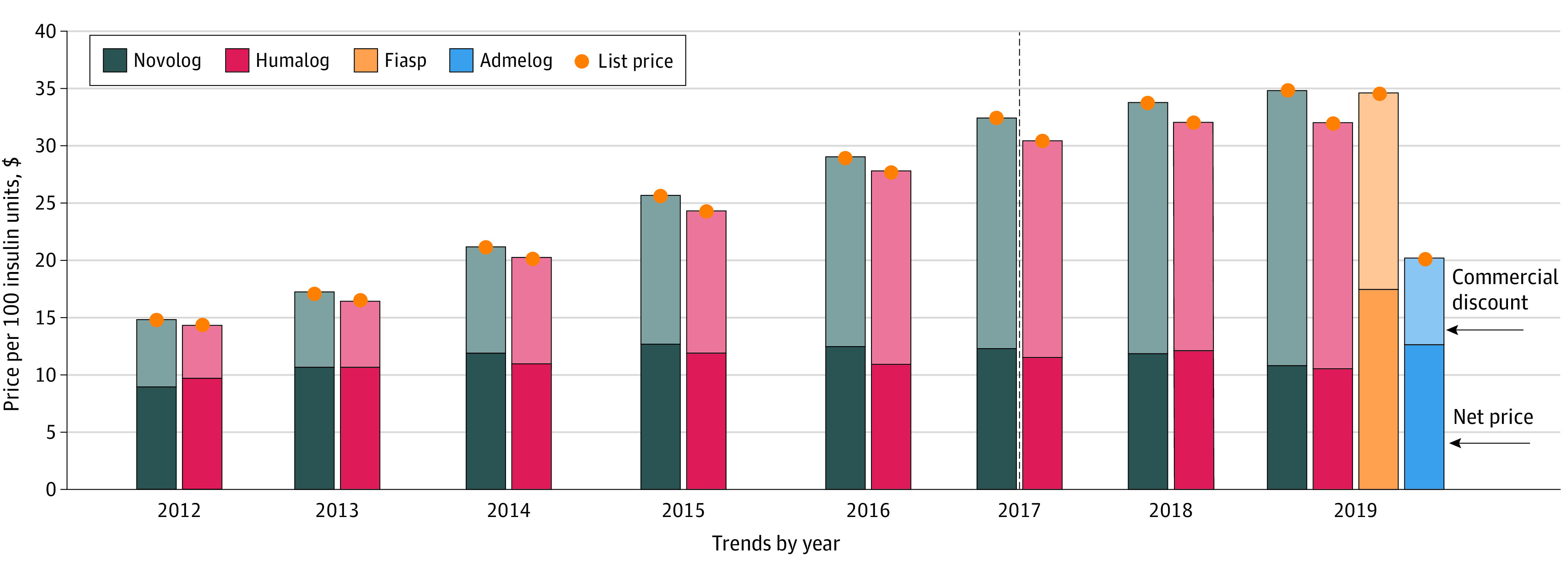
Trends in List Prices, Net Prices, and Discounts for Short-Acting Insulin Analogues List prices were estimated as mean reimbursement rates in Medicare Part D per 100 insulin units. Net prices were estimated as the difference between list prices and discounts and represent the prices faced by Part D and private plans after rebates. Fiasp was approved in September 2017 and insulin lispro (Admelog) in December 2017. As explained in the Methods, prices and discounts of new products are not reported for the first year after market entry (drugs approved in the first half of a calendar year) or 2 years after market entry (drugs approved in the second half of a calendar year) to mitigate how inventory variation may have affected the data, which is why data for Fiasp and insulin lispro are first reported in 2019. Outcomes are expressed per 100 insulin units. The dashed line indicates approval for Fiasp and insulin lispro.

The list price of the new faster-acting insulin aspart Fiasp ($34.75) closely matched the list price of the incumbent insulin aspart Novolog ($35.08; both manufactured by Novo Nordisk), but net price was higher for Fiasp ($17.36 for Fiasp vs $10.74 for Novolog) (Figure 2). Admelog, the insulin lispro successor to Humalog, had a 37% lower list price than Humalog in 2019 (Admelog, $20.39; Humalog, $32.18), but its net price was slightly higher (Admelog, $12.54; Humalog, $10.44). Trends in market share are shown in eFigure 2 in Supplement 1.

### Human Insulin Products

**Figure 3.**
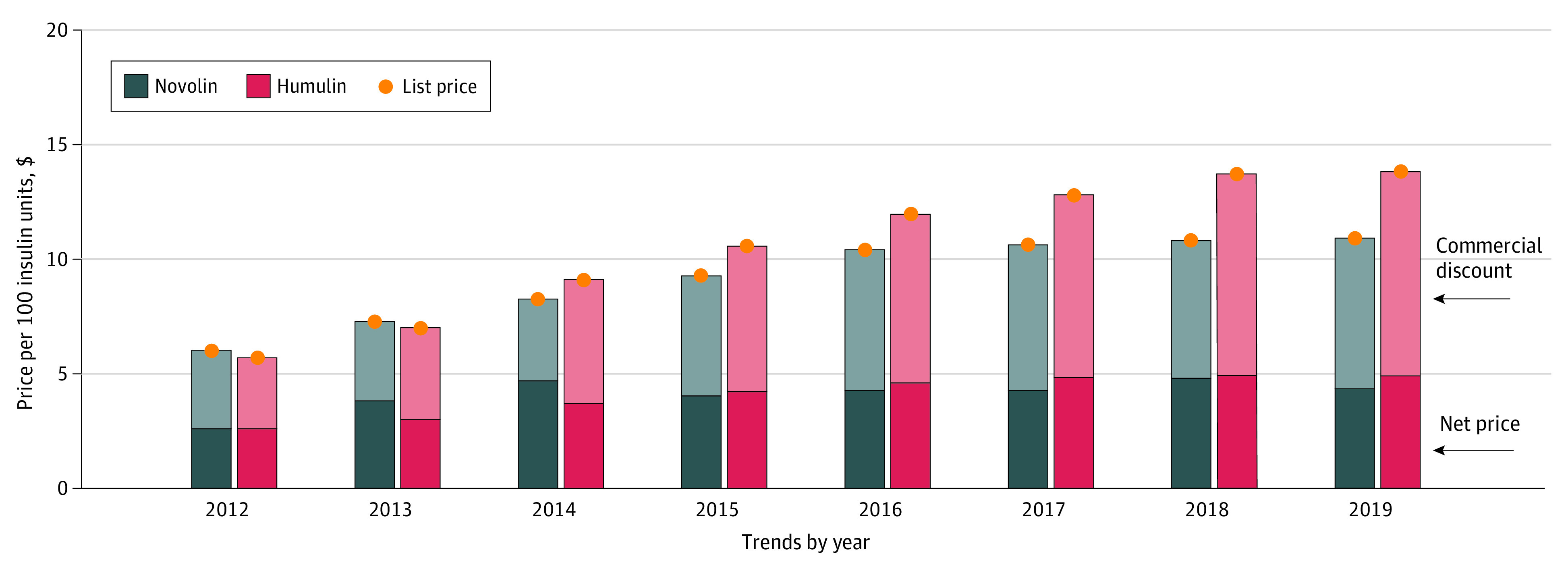
Trends in List Prices, Net Prices, and Discounts for Human Insulin Products List prices were estimated as mean reimbursement rates in Medicare Part D per 100 insulin units. Net prices were estimated as the difference between list prices and discounts and represent the prices faced by Part D and private plans after rebates. Outcomes are expressed per 100 insulin units.

The mean list and net price of human insulin products increased at an annual rate of 12.3% and 9.2% from 2012 to 2019, respectively (Table). Mean commercial discounts increased from 54.9% of list price to 63.1% during the same period. From 2012 to 2014, the net price of Novolin exceeded the net price of Humulin; however, the opposite was observed after 2015 (Figure 3).

## Discussion

To our knowledge, our study is the first to assess the net price of insulin products faced by commercial insurers, generating new insight into the relative competitiveness of the insulin market. We found large increases in net prices of insulins faced by payers from 2012 to 2015 before the approval of new products. The market entry of new insulin products was followed by increases in commercial discounts and subsequent decreases in net prices faced by payers.

The decreasing trends in net prices faced by payers observed for long-acting insulin products from 2014 to 2018 are consistent with the findings of previous studies that examined trends in prices across the entire insulin class.^18,20^ However, to our knowledge, our study is the first to report drug-specific estimates, which is important because the aggregation of insulin products with differing age since market entry, duration of action, and levels of competition obscures product-specific trends and may have led to substantial misestimation. Additionally, previous estimates of net prices do not account for Part D coverage gap discounts or for statutory discounts to the Medicaid program and the 340B Drug Discount program. As a result, they do not represent net prices faced by payers but rather manufacturer revenue per unit of product.^18,20,21^

Our method is a major contribution to the study of pharmaceutical prices because it is the first, to our knowledge, to isolate commercial discounts negotiated between manufacturers and payers from statutory discounts and coverage gap discounts. In doing so, it provides researchers and policy makers with additional tools to accurately characterize pricing and discounting trends in the pharmaceutical market, which are crucial for guiding policy-making efforts. Our estimated net prices are consistent with commercial discounts offered to larger payers disclosed by insulin manufacturers to the Senate Finance Committee, validating our approach. For example, Novo Nordisk disclosed that the mean commercial rebate on insulin detemir (Levemir) in 2015 was 40%, consistent with our findings.^22^ Novo Nordisk also disclosed a pricing strategy for insulin degludec (Tresiba) at a 27% premium to insulin detemir (Levemir), also consistent with our findings (Figure 1).^22^ Similarly, Sanofi disclosed that maximum recommended rebates for Lantus in 2015 were 36% for commercial insurance and 41% for Medicare Part D, consistent with our estimated mean commercial discount (Figure 1).^23^ Disclosures from Lilly indicated mean Humalog commercial rebates of 59% and Medicare rebates of 72% for 2016, consistent with our estimates (Figure 2).^24^ However, our estimates are mean values across all payers, and the Senate Finance Committee investigation revealed that discounts to individual payers varied widely according to the relative bargaining power and insured lives of each payer.^25^ Nevertheless, the consistency between our findings and reported rebates to the largest market players validates our approach.

Our findings are of relevance because policy making to address cost-related insulin access has been hampered by insufficient knowledge of the net price of insulin and its association with patient out-of-pocket costs, resulting in policy proposals that have been scored by the Congressional Budget Office as increasing the cost of insulin.^26^ Overall, our analysis confirms that insulin products are highly rebated and that net prices faced by payers increased at a slower pace than list prices from 2012 to 2019. Before competition, however, increasing discounts were not able to offset increases in list prices, meaning that list price growth resulted in higher net prices.

Our net price estimations revealed commercial strategies across insulin markets. Net prices of long-acting legacy insulin products Lantus and insulin detemir (Levemir) decreased after classwide competition owing to the introduction of biosimilar Basaglar, as well as Toujeo and insulin degludec (Tresiba), in 2015. At launch, Sanofi priced high-strength insulin glargine Toujeo at a lower net price than legacy insulin glargine Lantus even as it was reducing the net price of Lantus. This strategy, acknowledged by Sanofi to the Senate Finance Committee, aimed to encourage insurers to induce switches from Lantus to Toujeo, ostensibly to avoid automatic substitution of Lantus with forthcoming interchangeable biosimilars (first approved in 2021).^24^ Novo Nordisk priced insulin degludec (Tresiba) at a higher list and net price compared with legacy insulin detemir (Levemir), which likely reflects its ultralong duration of action unmatched by other products. However, by 2018, the net price of insulin degludec (Tresiba) had decreased below the net price of insulin detemir (Levemir) in 2014, suggesting intense competition in the crowded long-acting insulin market. The introduction of noninterchangeable insulin glargine Basaglar in 2015 was followed by decreases in net prices faced by payers across the entire class of long-acting insulin products. Although Toujeo has a lower net price than Basaglar, it has not captured as much market share as Basaglar (eFigure 1 in Supplement 1), which may reflect insurers’ concern about switching patients to a formulation that will not face biosimilar competition for a longer period. Together, the change in trajectory of net prices of long-acting insulin products after competition suggests that competition resulted in lower net prices, likely reducing overall insulin spending.

In the short-acting insulin market, we observed overall increases in the net prices of Novolog and Humalog before competition, suggesting that these 2 products were not aggressively competing on net price (Figure 2). These data are consistent with Lilly’s disclosed pricing strategy of matching Novo Nordisk’s list prices and rebates offerings.^24^ After the 2017 introduction of the insulin lispro Admelog, a noninterchangeable biosimilar for Humalog, and Fiasp, an improved formulation of Novolog also manufactured by Novo Nordisk, Novo Nordisk reduced the net price of Novolog to match that of Humalog. In 2019, net prices for both Novolog and Humalog decreased, resulting in a net price for Humalog below the net price for its biosimilar, Admelog. Fiasp, although priced at near-list-price parity with its precursor Novolog, maintained a premium net price. Annual net price increases of human insulin products, which did not experience competition, were greater than that observed in the long- and short-acting insulin analogue classes.

Our estimates also provide useful information to contextualize the March 2023 announcements by Eli Lilly, Novo Nordisk, and Sanofi of list price reductions on several of their insulin products. Eli Lilly announced a 70% price reduction for Humalog, Novo Nordisk a 75% price reduction for Novolog and a 65% price reduction for Novolin and insulin detemir (Levemir), and Sanofi a 78% price reduction for Lantus.^27,28^ These changes have been attributed to the forthcoming change to the calculation of Medicaid rebates that would have resulted in substantial penalties for historical price increases above the rate of inflation.^29^ These price reductions will bring list prices in line with our estimates of 2019 net prices faced by payers for each of these products, further confirming the accuracy of our estimates.

Researchers and policy makers have expressed concern that pharmacy benefit manager rebates by branded products may result in insurers’ preferring higher net-priced products with a rebate over lower net-priced products.^30^ Our data suggest that manufacturers are competing on net price but that a larger rebate may be a competitive advantage among similarly net-priced products. For instance, the net price of Lantus was lowered to match that of Basaglar even though Basaglar captured only 11% of market share. Insurer preference for Lantus may be due, in part, to its nearly 50% greater rebate. In the human insulin market, Humulin gained market share (eFigure 3 in Supplement 1) even though Novolin decreased its net price to be slightly lower than that of Humulin. Humulin uptake may reflect its larger rebates.

Across the 3 classes, even in the periods before new competition, net price growth was lower than others have estimated for branded pharmaceuticals overall,^31^ which suggests that pharmacy benefit managers have been effective in using formularies to reduce price growth even in duopoly markets. Pharmacy benefit managers also appear to have used new-brand competition to reduce net price growth before the introduction of interchangeable biosimilar products, as observed in the 2015 net price reductions among long-acting insulin analogues.

### Limitations

This study is subject to several limitations. First, unit sales data from SSR Health may not include all units sold, which would cause an overestimation of net price. However, this overestimation would be unlikely to affect the observed trends because any missing units are likely consistent from year to year. Second, our estimate of 340B sales in Medicare Part D may differ from the commercial market. Nevertheless, this difference would be unlikely to affect the observed trends. Third, our use of the mean pharmacy benefit manager rebate to estimate Medicaid best price likely understates the best price discount because that discount represents the greatest one available and not the mean. Given the consistency of our observed results with the disclosures by insulin manufacturers to the Senate Finance Committee, however, we believe that any differences attributable to these limitations are minor. Fourth, we did not account for patient assistance programs or discounts to the Department of Defense, the Department of Veterans Affairs, or other federal purchasers, which are therefore included under commercial discounts. This limitation will likely result in an overestimation of commercial discounts; however, it should not greatly affect the results because federal programs account for less than 5% of expenditures on prescription drugs.^32^ Fifth, our analysis was not able to differentiate how other market forces, such as political pressure on insulin prices, affected new product introduction.

## Conclusion

In the first longitudinal study, to our knowledge, to assess the net price of insulin products faced by payers, we observed that the introduction of new insulin products was followed by substantial discounting practices that lowered net prices.

## Notes

Supplement 1. **eAppendix.** Online-Only Methods **eFigure 1.** Trends in Market Share, Long-Acting Insulin Analogues **eFigure 2.** Trends in Market Share, Short-Acting Insulin Analogues **eFigure 3.** Trends in Market Share, Human Insulins
eReferences.

Supplement 2.
Data Sharing Statement
