# Supplementary material for: Estimated Changes in Insulin Prices and Discounts After Entry of New Insulin Products, 2012-2019
Source: JAMA Health Forum. 2023 Jun 16;4(6):e231430. doi: 10.1001/jamahealthforum.2023.1430 (PMC10276305; doi:10.1001/jamahealthforum.2023.1430)
Supplement: Supplement 1. — eAppendix. Online-Only Methods eFigure 1. Trends in Market Share, Long-Acting Insulin Analogues eFigure 2. Trends in Market Share, Short-Acting Insulin Analogues eFigure 3. Trends in Market Share, Human Insulins eReferences. [file jamahealthforum-e231430-s001.pdf]

Supplementary Online Content

Dickson S, Gabriel N, Gellad WF, Hernandez I. Estimated changes in insulin prices and discounts after entry of new insulin products, 2012-2019. *JAMA Health Forum*. 2023;4(6):e231430. doi:10.1001/jamahealthforum.2023.1430

eAppendix. Online-Only Methods
eFigure 1. Trends in Market Share, Long-Acting Insulin Analogues
eFigure 2. Trends in Market Share, Short-Acting Insulin Analogues
eFigure 3. Trends in Market Share, Human Insulins
eReferences.

This supplementary material has been provided by the authors to give readers additional information about their work.

© 2023 Dickson S et al. *JAMA Health Forum.*

eAppendix.

Overview of the Rationale for Discount Estimation

We provide a simplified explanation of the rationale followed for the derivation of commercial discounts, which are discounts negotiated between manufacturers and payers for Medicare Part D and group health insurance plans. Further details are described below and in a previous publication.[1]

(1) Total discounts = Gross sales – net sales

(2) Total discounts† = Medicaid discounts + 340B discounts + commercial discounts + coverage gap discounts

Solving for commercial discounts:

(3) Commercial discounts = Total discounts† – Medicaid discounts -340B discounts-coverage gap discounts

Substituting (1) into (3):

(4) Commercial discounts= Gross sales – net sales **–** Medicaid discounts -340B discounts-coverage gap discounts

- Gross sales can be estimated as list price * units
- Net sales are obtained from SSR Health
- Medicaid and 340B discounts are estimated as explained below.
- Coverage gap discounts are calculated using claims data from a 5% random sample of Medicare beneficiaries and extrapolated to the overall Medicare population.

Please note that in estimating commercial discounts, we account for the Medicaid Best Price provision. We perform alternative calculations based on whether the commercial discount sets Best Price, as described below and in our previous work.[1]

†Our methodology does not account for patient assistance programs or discounts to the Department of Defense, Department of Veterans Affairs, or other Federal programs, which are therefore included under commercial discounts.

Detailed Explanation of the Estimation of Commercial Discounts Based on the Best Price Provision

For branded drugs, Medicaid statutory rebates are the sum of the base rebate and the inflation rebate. The base rebate is calculated as the greater of 23% discount or the Best Price offered to any purchaser. The inflation rebate penalizes increases in prices since product launch that exceed general inflation. Our estimates of commercial discounts account for the Best Price provision as follows:

We subtract the inflation Medicaid and 340B inflation penalty discounts from the gross to net sales difference in (4):

(5) Gross sales – net sales- Medicaid inflation penalty- 340B inflation penalty-coverage gap discounts=**x**

We amortize the remaining discount **x** amount across the sum of commercial, Part D, Medicaid, and 340B program units to generate the estimated commercial discount.

- If the estimated commercial discount ≥23.1% of list price, then the assumption that commercial discount set Best Price holds, and the resulting estimate is the commercial discount.
- If the estimated commercial discount < 23.1% of list price, then the commercial discount does not trigger the Best Price provision. In this case, we re-estimate Medicaid and 340B discounts as the

© 2023 Dickson S et al. *JAMA Health Forum.*

sum of 23.1% of list price and inflation penalty. We subtract these re-calculated discounts to the Medicaid program and 340B discounts from the difference between gross and net sales and amortize the remaining amount across commercial and Part D units only. The resulting estimate is the commercial discount per unit.

The inflation penalty was calculated for each product and year as the difference between the list price and the inflation-adjusted launch price, following a published method.[2] Launch price data is available at the national drug code level and our analyses were conducted at the product level. To estimate an inflation-adjusted launch price at the product level, we calculated the inflation-adjusted launch price for each national drug code every year, and weighted by the relative utilization of each national drug code every year.

Accounting for Medicaid and 340B Discount Cap

Medicaid and 340B discounts are capped at 100% of the Average Manufacturer Price, in other words, the sum of the base discount and the inflation penalty cannot exceed the invoice price. We checked whether the sum of the estimated base rebate and inflation penalty exceeded the list price. For drug-year observations where it did, we recalculated the total amount of commercial discounts as follows:

(5') Gross sales – net sales- Medicaid and 340b units * list price-coverage gap discounts=**y**

Then, we amortized the remaining discount **y** amount across the sum of commercial and Part D units to generate the estimated commercial discount.

Input Parameters

Commercial discounts are estimated as a factor of gross sales, net sales, Medicaid discounts, and 340B discounts, as explained above. In this section we describe how we estimated each of these input parameters:

- Gross sales = total units * list price
    - Total units sold were obtained from Symphony Health. Symphony Health data has a 92% coverage of prescriptions dispensed across the US.[3] Total units were only available until the third quarter of 2019. Units for the fourth quarter of 2019 were projected using data from the previous quarters.
    - List price was estimated as the average reimbursement rate per insulin unit in the Medicare Part D dashboard.
- Net sales are obtained from SSR Health,[4] and represent sales reported by manufacturers for each product. Net sales are available at the overall branded level, as manufacturers do not separately report net sales at the national drug code level.
- Medicaid discounts= Medicaid discount per unit * Medicaid units.
    - Medicaid discounts per unit were estimated as explained above.
    - Medicaid units were obtained from the Medicaid spending dashboard.
- 340B discounts= 340B discount per unit * number of units subject to 340B discounts
    - 340B discount per unit =Medicaid discount per unit. Estimated as explained above
    - Number of units subject to 340B discounts= Medicare Part B 340B units + commercial 340B units (where commercial includes group health insurance plans and Medicare Part D)

© 2023 Dickson S et al. *JAMA Health Forum.*

  - Medicare Part B 340 Units = Medicare Part B Units * Proportion of Medicare Part B units subject to 340B discounts
    - Medicare Part B Units were obtained from the spending dashboard ‡
    - To estimate the proportion of Medicare Part B units subject to 340B discounts, we used Part B claims from a 5% random sample of Medicare beneficiaries and a previously published approach.[2] In brief, we extracted all Part B claims for the sample of drugs and identified those originating from 340B institutions by matching claims to the list of 340B institutions from the Health Resources & Services Administration (HRSA).[5] Carrier claims were matched using the national provider identifier and the address and outpatient claims using the Medicare Provider number.
  - Commercial 340B units = commercial units prescribed at 340B eligible entities* proportion of units prescribed at 340B eligible entities that are filled at 340B pharmacies.
    - Commercial units prescribed at 340B eligible entities were estimated by matching the Medicare Part D Prescriber Utilization File[6] to the 340B Covered Entity File for each year.[2]
    - To determine the proportion of 340B-Prescribed Units that were filled at 340B pharmacies, we first extracted pharmacy claims from a 5% random sample of Medicare beneficiaries that were prescribed by prescribers identified as 340B-eligible, using the methodology described above. Then, we calculated the proportion of these claims that were dispensed at 340B pharmacies by matching the dispensing pharmacy in each claim to the 340B Pharmacy File obtained from Health Resources & Services Administration.[5]

‡ The estimation of Part B units for each insulin product was limited by the availability of only two J codes for insulin (J1815 for insulin administered without a pump, and J1817 for insulin administered via pump). We allocated the insulin units reimbursed under J1815 across all insulin products available in a given year, following the breakdown of relative utilization of each product observed in Medicare Part D claims. Insulin units reimbursed under J1817 were distributed equally across the four insulin products used with pumps (Humalog, Novolog, Humulin, and Novolin). We were not able to allocate J1817 units based on relative utilization because we did not have access to durable medical equipment claims data, which captures a large share of insulin administered through pumps.

Adjustment for launch of Insulin Lispro Authorized Generic in 2019

In March 2019, Eli Lilly launched an authorized generic for Humalog (insulin lispro). Eli Lilly's 2019 company reports bundled sales for the branded and authorized generic in the reporting of net sales data, therefore, an approximation was necessary to subtract units and sales for the authorized generic from those of Humalog. This was necessary to avoid an overestimation of commercial discounts in 2019 for Humalog.

Based on the data reported in the Q4 2019 Eli Lilly financial report,[7] we assumed that 10% of the total 2019 units for insulin lispro were for the authorized generic and 90% for the branded product. We subtracted from the net sales figure for insulin lispro the amount corresponding to net sales for the authorized generic, assuming no discounts for the authorized generic (Net sales for Humalog = Combined net sales reported by Eli Lilly - 10% of units * list price of authorized generic). We also subtracted the units for the authorized generic from the total units figure, and re-estimated the subsequent parameters that were calculated on the basis of these estimates.

© 2023 Dickson S et al. *JAMA Health Forum.*

eFigure 1. Trends in Market Share, Long-Acting Insulin Analogues.

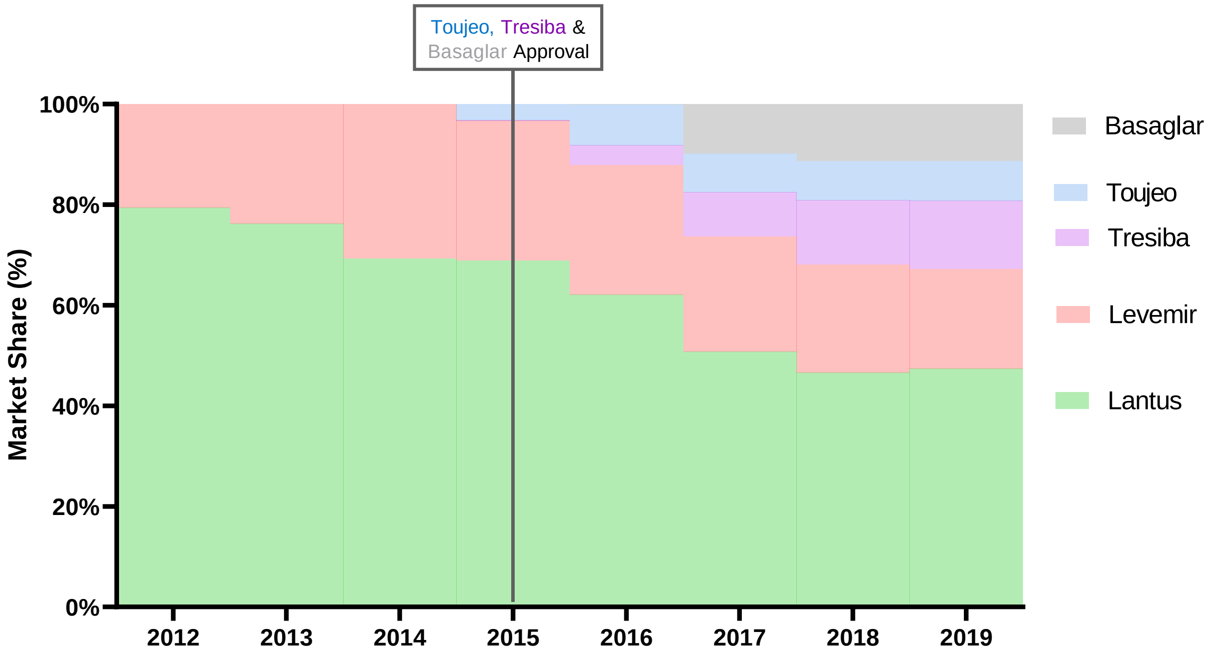

© 2023 Dickson S et al. *JAMA Health Forum*.

eFigure 2. Trends in Market Share, Short-Acting Insulin Analogues.

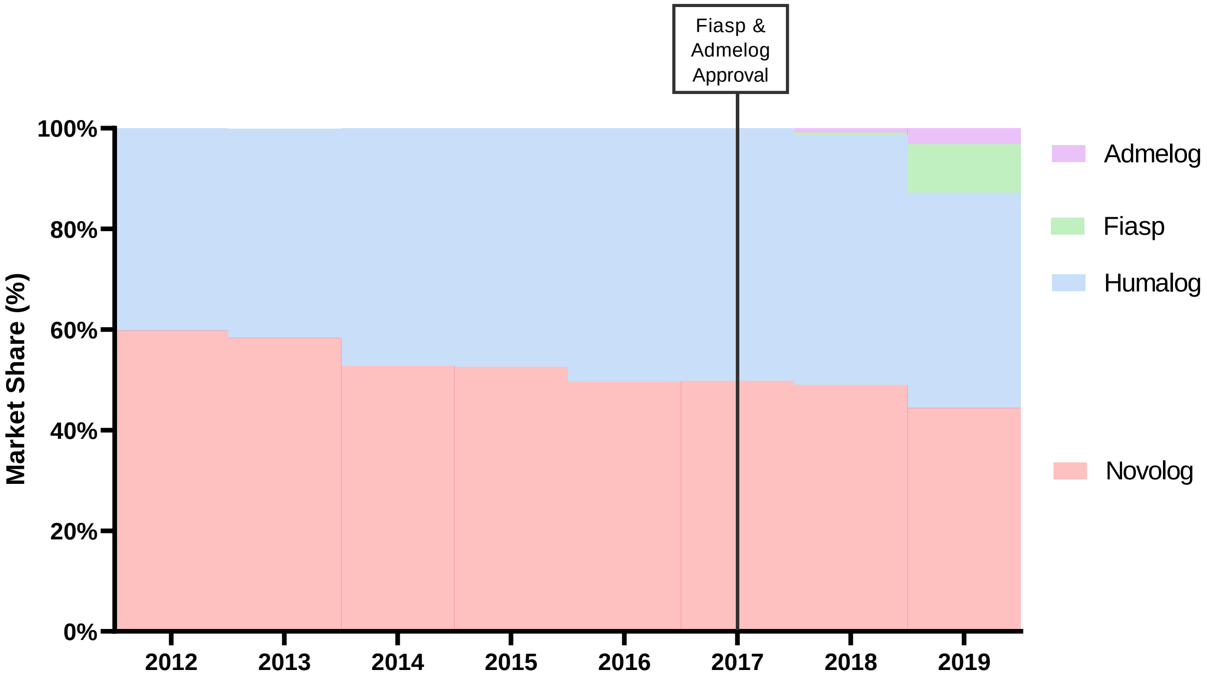

© 2023 Dickson S et al. *JAMA Health Forum*.

eFigure 3. Trends in Market Share, Human Insulins.

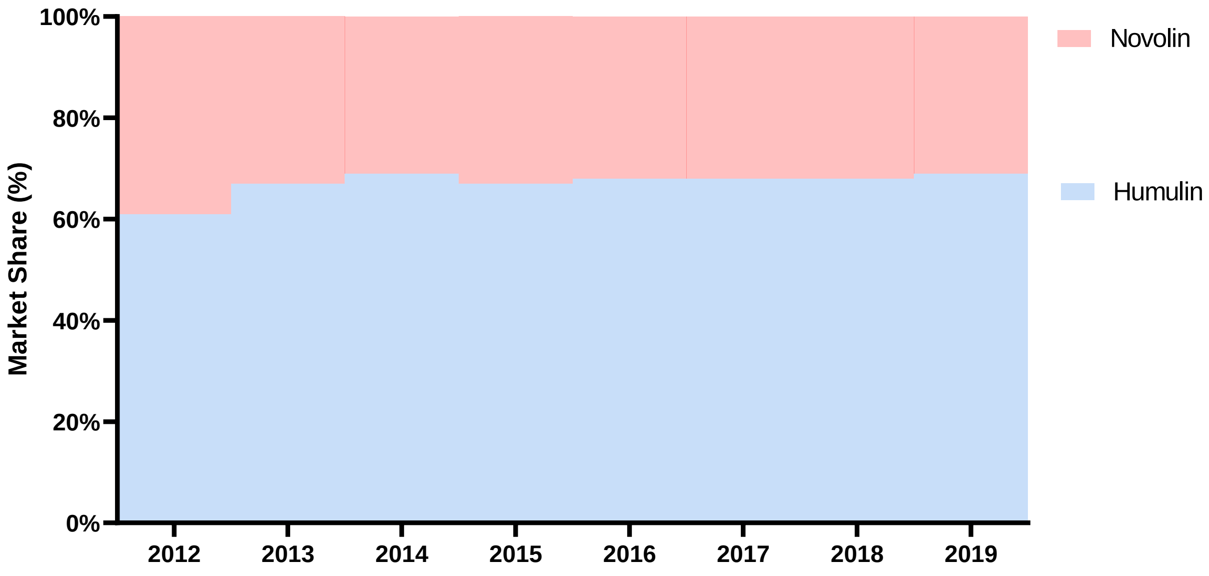

© 2023 Dickson S et al. *JAMA Health Forum.*

eReferences.

1. Dickson S, Gabriel N, Hernandez I. Estimated Changes in Price Discounts for Tenofovir-Inclusive HIV Treatments Following Introduction of Tenofovir Alafenamide. *AIDS 2022 doi: 101097/QAD0000000000003401*.

2. Dickson S, Gabriel N, Gellad W, Hernandez I. Reduction in Medicaid rebates paid by pharmaceutical manufacturers for outpatient injected, inhaled, infused, implanted, or instilled drugs: The 5i loophole. *J Health Polit Policy Law*. Published online July 14, 2022. doi:10.1215/03616878-10041219

3. Symphony Health Data Overview. Accessed September 14, 2022. https://symphonyhealth.com/what-we-do/view-health-data

4. SSR Health. Accessed September 14, 2022. https://www.ssrhealth.com/dataset/

5. Health Resources & Services Administration. 340B Drug Pricing Program. Accessed September 14, 2022. https://www.hrsa.gov/opa

6. Medicare Provider Utilization and Payment Data: Part D Prescriber. Accessed September 14, 2022. https://data.cms.gov/provider-summary-by-type-of-service/medicare-part-d-prescribers

7. Eli Lilly. Lilly Reports Strong Fourth-Quarter and Full-Year 2019 Financial Results, Updates 2020 Guidance for Pending Dermira Acquisition. Published January 30, 2020. Accessed October 6, 2022. https://investor.lilly.com/static-files/f2ad4e6f-ca7b-4d45-876d-0fd12995aab0

8. IQVIA Institute Report. Medicine Spending and Affordability in the U.S. Accessed September 14, 2022. https://www.iqvia.com/insights/the-iqvia-institute/reports/medicine-spending-and-affordability-in-the-us

© 2023 Dickson S et al. *JAMA Health Forum.*
