# Supplementary material for: Estimated Changes in Insulin Prices and Discounts After Entry of New Insulin Products, 2012-2019
Source: JAMA Health Forum. 2023 Jun 16;4(6):e231430. doi: 10.1001/jamahealthforum.2023.1430 (PMC10276305; doi:10.1001/jamahealthforum.2023.1430)
Supplement: Supplement 2. — Data Sharing Statement [file jamahealthforum-e231430-s002.pdf]

Data Sharing Statement

Dickson. Estimated Changes in Insulin Prices and Discounts After Entry of New Insulin Products, 2012-2019. *JAMA Health Forum*. Published June 16, 2023. doi:10.1001/jamahealthforum.2023.1430

Data
Data available: No

Additional Information
Explanation for why data not available: Data are propietary
